# A setup for millisecond time-resolved X-ray solution scattering experiments at the CoSAXS beamline at the MAX IV Laboratory

**DOI:** 10.1107/S1600577522000996

**Published:** 2022-02-16

**Authors:** Oskar Berntsson, Ann E. Terry, Tomás S. Plivelic

**Affiliations:** a MAX IV Laboratory, Lund University, Lund, Sweden

**Keywords:** beamline, time-resolved X-ray solution scattering, time-resolved SAXS

## Abstract

The function of biomolecules is tightly linked to their structure, and changes therein. Time-resolved X-ray solution scattering has proven a powerful technique for interrogating structural changes and signal transduction in photoreceptor proteins. However, these only represent a small fraction of the biological macromolecules of interest. More recently, laser-induced temperature jumps have been introduced as a more general means of initiating structural changes in biomolecules. Here we present the development of a setup for millisecond time-resolved X-ray solution scattering experiments at the CoSAXS beamline, primarily using infrared laser light to trigger a temperature increase, and structural changes. We present results that highlight the characteristics of this setup along with data showing structural changes in lysozyme caused by a temperature jump. Further developments and applications of the setup are also discussed.

## Abstract 2

A new experimental setup for millisecond time-resolved X-ray solution scattering has been developed and commissioned at the CoSAXS beamline at the MAX IV synchrotron. Results from T-jump experiments induced by infrared laser pulses in lysozyme in solution are presented for validation.

## 1. Introduction

Biological macromolecules such as proteins or nucleic acids are structurally dynamic molecules. This means that to fully understand their function under various conditions it is important that they are studied using techniques which allow structural changes to take place.

Small-angle X-ray scattering (SAXS) is a technique that can simultaneously probe structure at multiple length scales, from protein–protein interactions to the internal structure of a protein. Modern day synchrotrons are capable of delivering a very high photon flux, which enables experiments with a high time-resolution to be conducted. Time-resolved SAXS, or, more generally, time-resolved X-ray solution scattering (TR-XSS), is highly sensitive to structural changes, capable of detecting large-scale changes in the global structure, as well as the escape of a small ligand, as in the case of carbon monoxide escaping myoglobin (Cho *et al.*, 2010 ▸).

TR-XSS requires that some sort of perturbation initiates a structural change in the sample. Structural changes can be caused by rapidly mixing solutions to cause, for example, the addition of a ligand, or a change in temperature. Such setups can be found, for example, at the BioCAT (Advanced Photon Source, APS) (Graceffa *et al.*, 2013 ▸) or P12 (Petra III) (Blanchet *et al.*, 2015 ▸; Josts *et al.*, 2020 ▸) beamlines.

Light has the possibility of triggering reactions with unmatched temporal and high spatial precision. As such, combining laser-induced structural changes with X-ray scattering might be appealing. Photoactive proteins display a conformational change in response to light stimulus, and thus a laser pulse of appropriate wavelength can be used to induce a structural change in such samples (Andersson *et al.*, 2009 ▸; Malmerberg *et al.*, 2011 ▸; Kim *et al.*, 2012*a* ▸; Cho *et al.*, 2016 ▸; Björling *et al.*, 2016 ▸; Berntsson *et al.*, 2017*a* ▸,*b* ▸, 2019 ▸). Time-resolved experiments inducing protein structural changes with a laser trigger were first carried out at beamline ID09 (European Synchrotron Radiaion Facility) (Wulff *et al.*, 2003 ▸; Cammarata *et al.*, 2008 ▸; Andersson *et al.*, 2009 ▸) (ps–ms resolution) and have since been carried out at, for example, BioCARS (APS) (Graber *et al.*, 2011 ▸) (ps–ms resolution) and cSAXS (Swiss Light Source, SLS) (Westenhoff *et al.*, 2010 ▸) (primarily tens of ms resolution).

Only a minority of proteins, however, are directly photoactive. For some proteins a ligand can be dissociated from the protein by means of light (Cammarata *et al.*, 2008 ▸; Cho *et al.*, 2010 ▸; Kim *et al.*, 2012*b* ▸). In other cases, the ligand is released from a photolabile cage in response to light (Klán *et al.*, 2013 ▸), and thus becomes available to the protein (Ravishankar *et al.*, 2020 ▸; Orädd *et al.*, 2021 ▸; Josts *et al.*, 2018 ▸) or changes the environment (Rimmerman *et al.*, 2018 ▸, 2019 ▸). Because much of biochemistry is thermally driven, functionally relevant conformational changes can also be triggered by changes in temperature. Infrared (IR) light with a wavelength of ∼1450 nm is readily absorbed by water (Wang *et al.*, 2013 ▸), resulting in a temperature increase. Thus, by using IR lasers to trigger temperature jumps (T-jumps) a more general method for studying the structural dynamics of biomolecules is available (Rimmerman *et al.*, 2017 ▸; Thompson *et al.*, 2019 ▸; Henry *et al.*, 2020 ▸).

In this contribution we present a setup for performing T-jump TR-XSS experiments at the CoSAXS beamline at the MAX IV Laboratory. By using detectors with a high frame rate, in a SAXS (for monitoring protein dynamics)/WAXS (for monitoring solvent dynamics) configuration, in combination with an IR laser pulse, we are able to record data with a 2 ms time-resolution. Future prospects of the setup are also discussed.

## 2. Experimental setup

### 2.1. The CoSAXS beamline

CoSAXS is the coherent and SAXS beamline placed at the diffraction-limited 3 GeV storage ring at the MAX IV Laboratory (Plivelic *et al.*, 2019 ▸). The beamline comprises a Si(111) horizontally deflecting double-crystal monochromator followed by two pairs of mechanically bendable flat mirrors in Kirkpatrick–Baez configuration. This configuration enables independent focusing in both horizontal and vertical directions. The X-ray focus may be at the sample, or on the SAXS detector. The beamline also provides simultaneous WAXS detection capabilities. CoSAXS is a hard X-ray beamline with a tunable energy from 4 to 20 keV and a bandwidth Δ*E*/*E* = 2 × 10^−4^ and a photon flux of 10^12^–10^13^ photons s^−1^.

### 2.2. Sample environment and scattering geometry

**Figure 1:**
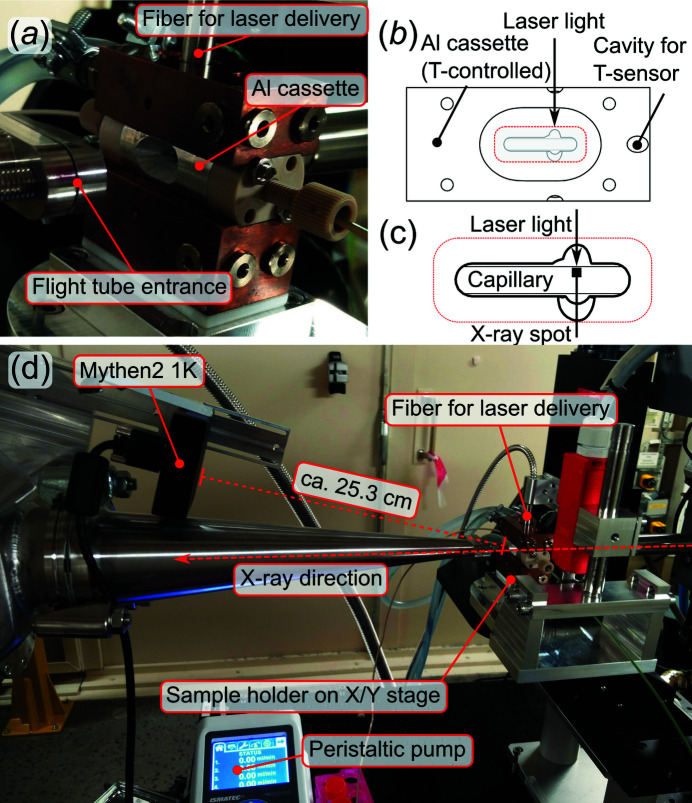
Flow cell and environment at the CoSAXS experimental table. (*a*) The flow cell capillary holder. (*b*) The aluminium cassette of the capillary holder and (*c*) a close up indicating where the X-rays and laser are directed. The size of the laser spot is ∼0.9 mm FWHM and the X-ray spot is ∼160 µm × 140 µm. (*d*) The setup at the sample table. Fresh sample is delivered through PEEK tubing by a peristaltic pump. The irradiating laser light arrives through a fiber. The sample holder is placed on an *X*/*Y* stage ∼25 cm from the WAXS 1D detector and 2 m from the 2D SAXS detector (outside photograph).

The time-resolved sample environment at CoSAXS is primarily developed to study proteins in aqueous solutions. The temperature-controlled flow cell [Figs. 1 ▸(*a*)–1(*c*)], based on the design of the BioSAXS flow cell at the SWING beamline in SOLEIL (Thureau *et al.*, 2021 ▸), consists of a 1.5 mm inner-diameter quartz capillary, with a 10 µm wall thickness. The temperature of the flow cell is controlled by an external water bath that can be set to temperatures in the range 8–80°C. A temperature sensor is attached to the aluminium cassette holding the capillary. The temperature reading at this position is considered to represent the sample baseline temperature. The flow cell is positioned on an *X*/*Y* stage [Fig. 1 ▸ (*d*)]. Sample is delivered from a reservoir via peristaltic and PEEK tubing, using a peristaltic pump.

A dual detector system is used. An Eiger2 4M (Dectris) is positioned in a 17 m long evacuated flight tube and covers SAXS angles (∼6 × 10^−4^ < *q* < 0.6 Å^−1^, where *q* = 4πsin(θ)/λ, with 2θ the scattering angle and λ the X-ray wavelength). The SAXS detector can be moved closer to or further away from the sample position to study different length scales in the samples. A Mythen2 1K (Dectris) is placed in air, ∼25 cm from the sample, and covers WAXS angles (∼1.4 < *q* < 2.5 Å^−1^). The WAXS detector, and WAXS range, is of particular importance for T-jump experiments, as will be described in detail later. The sample cell is placed in air, about 2 cm from the evacuated flight tube, where a 50 µm-thick Kapton film covers the entrance.

### 2.3. Time-resolved optical pump/X-ray probe experiments

The experimental setup at CoSAXS is conceptually similar to the time-resolved experiments which have been set up at the cSAXS beamline at the SLS (Westenhoff *et al.*, 2010 ▸) but uses an IR laser to trigger rapid T-jumps. The synchronization of detector readout and laser pulse (arrival and duration) is controlled electronically via the PandABox FPGA (Zhang *et al.*, 2017 ▸). In the present configuration, the time-resolution of the experiment is determined primarily by the frame rate of the detectors, and/or the laser pulse duration (which correlates with the desired T-jump). The Eiger2 4M can be operated at 500 Hz, and the Mythen2 1K can be operated at 1 kHz. This means that structural changes can be monitored with a 2 ms time-resolution. While the SAXS detector can be read out in about 3 µs, the WAXS detector requires approximately 90 µs. This means that, when operating the detectors at 500 Hz, each frame on the detector corresponds to the sum of scattering collected during 1.9 ms.

While this contribution focuses on T-jumps induced by an IR laser pulse, in principle any light source capable of receiving a TTL trigger pulse may be used. In the present configuration, the time-resolved setup makes use of a LuOceanP2 (Lumics) 1470 nm, 50 W, continuous wave (CW) laser. Wavelengths around 1450 nm are absorbed by the O–H bond in water (Wang *et al.*, 2013 ▸). The laser can be modulated by a TTL gate pulse and the output power scales linearly with the pulse duration down to ∼1 ms. A train of secondary pulses can also be triggered in order to maintain the initial T-jump. The laser is fiber coupled and the light is guided by a 200 µm, 0.22 NA fiber (where NA is numerical aperture). The other end of the fiber is physically attached to the flow cell, positioning the tip of the fiber within a few mm of the capillary wall.

**Figure 2:**
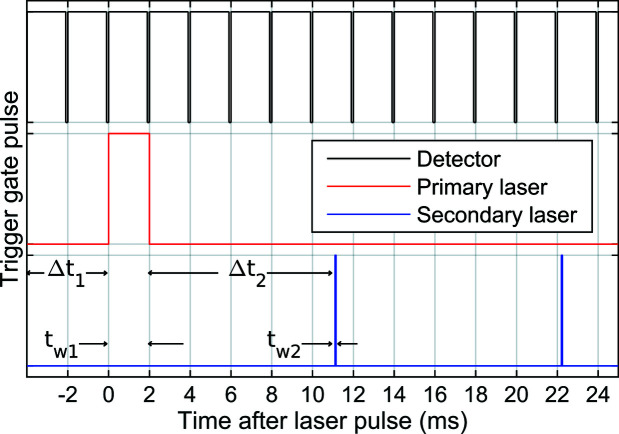
Trigger signals. When the trigger gate pulse is high, the detector acquires data or the laser fires. Trigger diagrams are vertically offset for clarity.

Typical TR-XSS data acquisition at CoSAXS consists of a scan with alternating *laser off* and *laser on* steps. Each step comprises a number of frames (or images). A scan typically both begins and ends with a *laser off* step, for reasons which will be explained in the next section. The sample remains static in the X-ray beam over the course of a step. Between steps the old sample is flushed out and new fresh sample is added automatically. Fig. 2 ▸ depicts a trigger diagram showing how detector acquisition, primary laser trigger and train of secondary laser triggers might appear during a *laser on* step. X-ray exposure and detector data acquisition are started at the same time. After some delay (Δ*t*_1_) the primary laser pulse arrives. The primary laser pulse has some duration (*t*_w1_). If secondary pulses are used, these will arrive after yet another delay (Δ*t*_2_), and be of a certain duration (*t*_w2_).

#### 2.3.1. Data analysis

Much of the sensitivity of the TR-XSS methodology is because of the use of a *difference* scattering protocol [Δ*I*(*q*)]. Compared with a conventional SAXS experiment where separate scattering patterns are taken of the sample and buffer in consecutive measurements, here the *laser off* measurements are interleaved and directly subtracted from the *laser on* data. In practice, this means that a scan consists of several steps where every alternate step is *laser on* and every other step is *laser off*. Neighbouring *laser off* steps are then averaged and subtracted from every *laser on* step. Averaging and subtraction is performed on a frame-by-frame basis, *i.e.* frame *n* of steps *m* and *m* + 2 (where *m* is odd) are averaged together and subtracted from frame *n* of step *m* + 1. In short, this puts the focus on what is different between the two measurements.

Before subtracting *laser off* data from *laser on* data, the scattering may be normalized to account for various experimental drifts, such as variations in incident photon flux and/or synchrotron ring current variations during the experiment, typically about a 10% variation in incident intensity between synchrotron refill injections which are every ten minutes. It is possible to normalize against transmitted intensity, but for aqueous samples it is also possible to normalize against the water scattering. This is because, when heated, the water scattering signal shows isosbestic points at *q* ≃ 1.5 Å^−1^ and *q* ≃ 2.1 Å^−1^ (Cammarata *et al.*, 2008 ▸; Kim *et al.*, 2012*b* ▸). In this *q*-range the protein contribution to the scattering is minuscule. Normalizing against the water scattering also allows us to correct for small variations in sample thickness.

The difference scattering data will not only depend on the structural change of the protein but also on the structural change of the solvent. While the structural change of water is almost only visible for scattering at *q* > 1 Å^−1^ it is still advisable to subtract the solvent contribution. This is done by separately recording data for a T-jump of the pure solvent, scaling this to the solvent + solute data at *q* ≃ 2 Å^−1^ and thereafter subtracting this contribution.

Rapid feedback is essential for guiding a TR-XSS experiment and at CoSAXS the (SAXS) data are radially integrated in real time, making 1D curves readily available. We also offer a small collection of MATLAB scripts specifically developed to be modular and easy to use for the online analysis of TR-XSS data (Berntsson, 2021 ▸).

#### 2.3.2. T-jump TR-XSS experiments on lysozyme

To showcase the capabilities of the time-resolved setup, TR-XSS experiments on lysozyme were performed. Lysozyme (chicken egg-white) was purchased from Sigma-Aldrich (catalogue No. L7651) and used without further purification. The protein was dissolved in a buffer solution (HEPES 20 m*M*, pH 7.2, 5% v/v glycerol) to a final concentration of ∼40 mg ml^−1^ and filtered through a 0.2 µm filter before use. The low ionic strength of the solution serves to promote repulsive inter­actions, thus preventing aggregation (Arai & Hirai, 1999 ▸), and the glycerol mitigates radiation damage (Kuwamoto *et al.*, 2004 ▸; Brooks-Bartlett *et al.*, 2017 ▸). In order to assess whether IR radiation absorbed by the protein rather than the aqueous solvent affected the scattering, experiments were also performed with lysozyme dissolved in a buffer prepared with deuterated water, since this is virtually transparent to light with a wavelength of 1470 nm. Note, however, that the other constituents of this buffer are protonated.

The X-ray energy was set to 12.4 keV and the X-ray spot size at the sample position was ∼160 µm × 140 µm. The sample-to-detector distance was 2.08 m (SAXS) and 25.3 cm (WAXS), resulting in accessible *q*-ranges of ∼0.01 < *q* < 0.6 Å^−1^ and 1.45 < *q* < 2.5 Å^−1^, respectively. The centre of the X-ray beam struck the capillary ∼0.5 mm from the top wall, where the laser is aimed. The sample was delivered from its reservoir via 0.76 mm inner-diameter PEEK tubing to the 1.5 mm inner-diameter quartz capillary at the flow cell. The baseline temperature of the flow cell was set to 20, 30 or 40°C. These temperatures are chosen because they are physiologically relevant and well below the unfolding temperature of lysozyme at ∼65°C (Arai & Hirai, 1999 ▸). What we intend to probe is thus not the (un)folding but rather thermally induced structural dynamics as lysozyme adapts to a new temperature.

Each scan consisted of alternating *laser off* and *laser on* steps. The detectors are read out at 500 Hz (1.9 ms acquisition, 100 µs readout) throughout each 4 s step, resulting in 2000 frames per step. Between each step the sample was replenished. During each *laser on* step a T-jump was triggered, 1 s after data acquisition commenced, by a 2 ms-long IR laser pulse. The T-jump is then maintained by a train of secondary pulses, each 50 µs long, fired at 90 Hz, for 3 s. The data were radially integrated (SAXS) and normalized to the scattering at *q* ≃ 1.5 Å^−1^.

## 3. Results

### 3.1. Calibration of the T-jump

To monitor the magnitude and evolution of the T-jump, the WAXS signal (1.45 < *q* < 2.5 Å^−1^) is of utmost importance since it is the reorientation of water molecules upon heating that provides an internal reference of the T-jump. By measuring the scattering of a sample of water or buffer at different temperatures and subtracting these to obtain difference scattering curves (note that this is not the same procedure as when difference scattering is recorded), it is possible to calibrate the magnitude of the T-jump. As can be seen in Fig. S1 of the supporting information, there is an almost perfect linear relationship between signal amplitude and temperature difference. Using the same baseline/reference temperature for the T-jump and the calibration measurements ensures the most accurate calibration.

### 3.2. WAXS signal-to-noise

**Figure 3:**
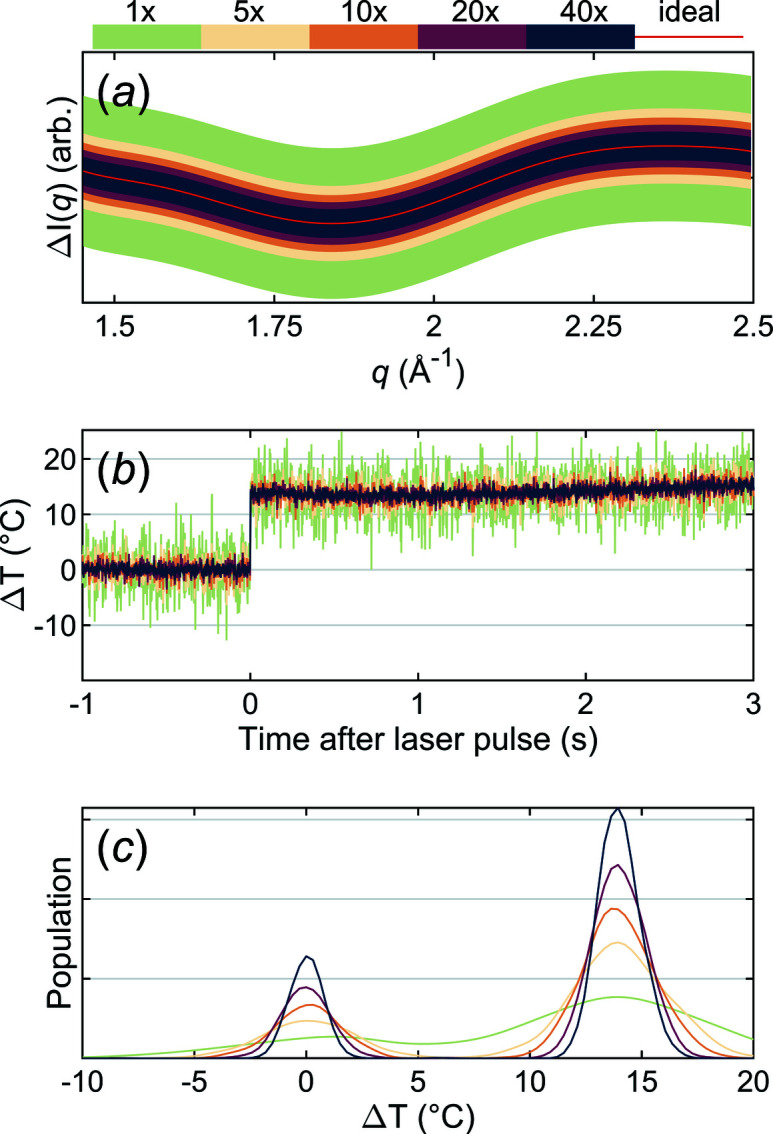
(*a*) The approximate signal-to-noise in the difference scattering signal in the WAXS range for different amounts of X-ray exposure, where 1× refers to a single frame (2 ms exposure), 5× to the average of five such frames, *etc*. (*b*) The estimated T-jump for different amounts of acquired data for a single step, the average of five steps, *etc*. (*c*) The data from (*b*) as a density distribution.

The current setup at CoSAXS uses a Mythen2 1K detector to cover the WAXS range and record the scattering of the solvent. While this detector is simple to use and affordable, the question is whether a detector consisting of a single array of 50 µm-wide pixels, placed in air, can be used to monitor the change in scattering in the WAXS region caused by modest T-jumps. To investigate this we assessed the data quality for different amounts of X-ray exposure. Fig. 3 ▸ shows the signal-to-noise for different levels of X-ray exposure of a sample of water. As can be seen in Fig. 3 ▸(*a*), a single difference curve [2 ms X-ray exposure, 10^12^ photons s^−1^ (with *laser on*) minus the average of 2 × 2 ms X-ray exposure (with *laser off*)] shows significant noise and will hardly be detected. After averaging five such frames, a more clear difference signal will be observed. The signal-to-noise of a single frame provides some idea of the precision with which the T-jump can be determined, but it is also useful to look at the set of frames corresponding to a full 4 s data acquisition step [Figs. 3 ▸(*b*)–3(*c*)]. In panel (*b*) we assess how much of the idealized curve from panel (*a*) is present at the different timepoints [Δ*I*(*q*, *t*) = *c*(*t*)Δ*I*_heat_(*q*), where *c* is a scalar that shows how the calibration difference heat data, Δ*I*_heat_(*q*), has to be scaled to fit the difference scattering data, Δ*I*(*q*, *t*)]. Panel (*c*) shows the same data as panel (*b*) but as a density distribution. These data clearly show that after a single step it is already possible to detect a T-jump. While the T-jump in the experiments described here was ∼15°C, after averaging five steps the magnitude of a T-jump as low as 5°C can be reliably determined.

### 3.3. Magnitude, temporal evolution and spatial profile of the T-jump

**Figure 4:**
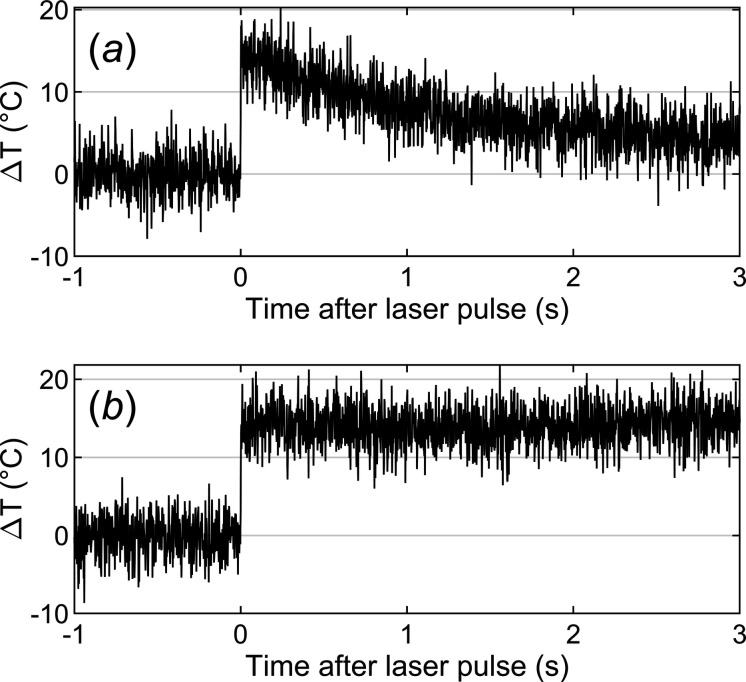
Temperature-dependent difference scattering signal. (*a*) The decay back to the base temperature, following a single 2 ms laser pulse. (*b*) The T-jump is maintained by supplying a train of secondary pulses, in this case 50 s-long pulses are triggered at 90 Hz over 3 s following the initial T-jump.

Within ∼100 ms of the T-jump the temperature begins to decay towards the baseline temperature of the flow cell [Fig. 4 ▸(*a*)]. However, by tuning the duration and frequency of a train of secondary pulses, it is possible to maintain the T-jump for several seconds. In this case, a train of 50 µs-long pulses, fired at 90 Hz, was capable of maintaining a stable T-jump for about 3 s [Fig. 4 ▸(*b*)].

**Figure 5:**
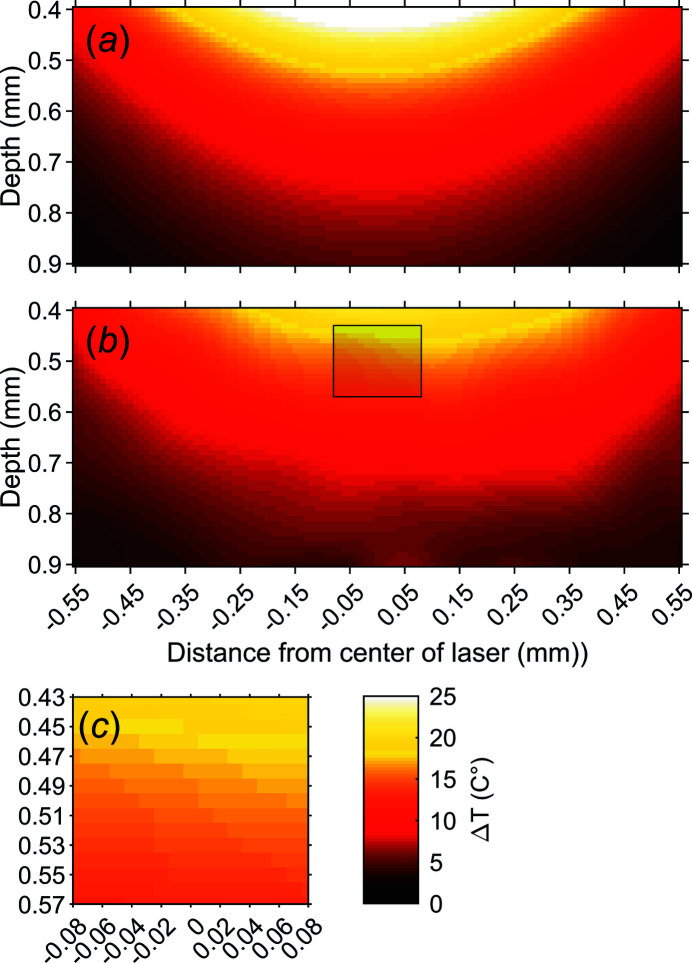
The spatial profile of the T-jump as seen by the X-ray beam. (*a*) Simulated T-jump and (*b*) observed T-jump over a section of the capillary. The grey box marks the approximate size of the X-ray spot and where it would be positioned during regular data acquisition. (*c*) Zoomed-in view of the grey box, showing the T-gradient of the probed volume.

The magnitude and spread of the T-jump was initially modelled by complex fluid dynamics (CFD) simulations. The simulations were performed using *COMSOL* 5.5 (COMSOL, 2019 ▸). In the simulations, the laser spot is simulated as a Gaussian supply of heat, with FWHM of 0.9 mm. Heating was achieved by adding 50 W over 2 ms, to a cylindrical body of water, with a diameter of 1.5 mm and initial temperature of 20°C. The absorption coefficient of 1470 nm light in water is ∼30 cm^−1^ (Kou *et al.*, 1993 ▸). Part of the light (∼5%) is reflected at the surface of the water cylinder. The predicted T-jump observed by the X-rays, 10 ms after heating, is depicted in Fig. 5 ▸(*a*).

The spatial profile T-jump was also determined empirically. A grid with a spacing of 100 µm in the horizontal and vertical direction was defined and the apparent T-jump measured in the grid points and values in between were interpolated [Fig. 5(*b*) ▸]. The usual position of the X-ray spot (160 µm × 140 µm) during the experiment is marked by a grey box. In Fig. 5 ▸(*c*) we can see that at this point the X-rays observe a T-jump of ∼16°C, but that there is some inhomogeneity within the probed volume. By carefully choosing the positioning of the X-ray spot, a higher- or lower-amplitude T-jump can be observed. The simulated and measured T-jump agree very well although the simulation displays a somewhat steeper temperature gradient than what is observed during the experiment. Over the mapped region, the simulated T-jump is 0.6 to 1.4 times that of the experimentally determined one. This level of accuracy is expected based on the simplified model used by *COMSOL*.

The depth into the capillary, that is, the distance from the top edge of the capillary, should be >400 µm [Fig. 1 ▸(*b*)]. Measurements closer to the top of the capillary show significant artefacts in the scattering data, caused by the quartz capillary.

### 3.4. T-jump TR-XSS measurements on lysozyme

**Figure 6:**
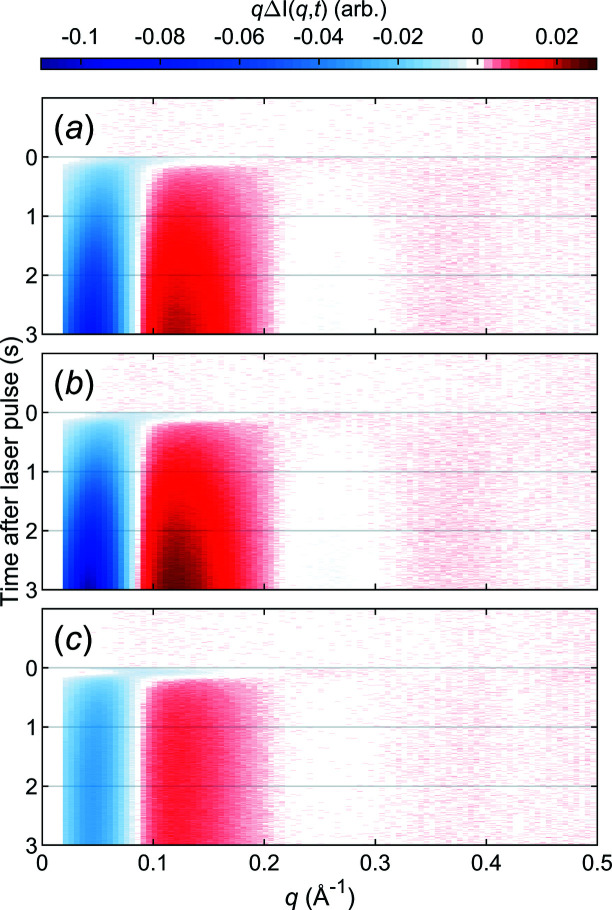
Data from a T-jump TR-XSS experiment on lysozyme. (*a*) Heat subtracted data in the SAXS range for a T-jump on lysozyme starting at 20°C. (*b*) Heat subtracted data in the SAXS range for a T-jump on lysozyme starting at 30°C. (*c*) Heat subtracted data in the SAXS range for a T-jump on lysozyme starting at 40°C.

T-jump TR-XSS data for lysozyme, with the solvent heating contribution subtracted, are shown in Fig. 6 ▸. The data are the result of ∼50–60 repetitions (50–60 *laser on* steps and 60–72 *laser off* steps). The T-jump was measured by comparing it with static data (Fig. S1) and is displayed in Table 1 ▸. Prominent features in the low-*q* region are consistent with large-scale structural changes, as *q* is related to characteristic distances, *d*, as *q* = 2π/*d*. The reduced intensity in the SAXS region can be attributed to a more expanded protein (Rimmerman *et al.*, 2017 ▸; Cho *et al.*, 2010 ▸). A more elaborate structural interpretation is outside the scope of this contribution.

**Figure 7:**
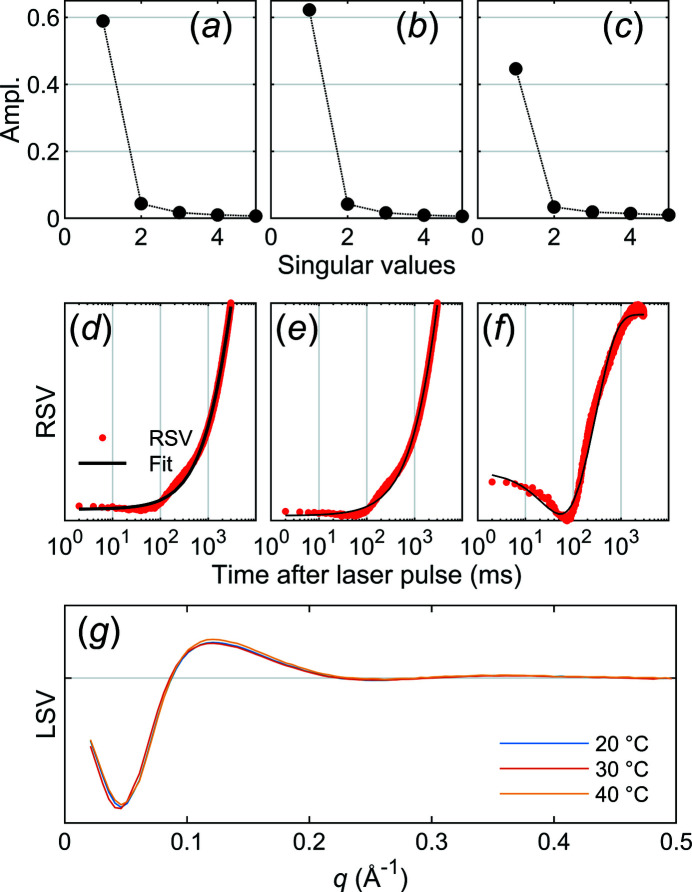
SVD analysis of lysozyme data. (*a*–*c*) Relative amplitude of the first five singular values from an SVD analysis of lysozyme data from a T-jump starting from 20°C (*a*), 30°C (*b*) and 40°C (*c*). (*d*–*f*) The first right singular vector (RSV), the temporal evolution of the signal, 20°C (*d*), 30°C (*e*) and 40°C (*f*). (*g*) The first left singular vector (LSV) at the three temperatures.

The signal develops over time and a singular value decomposition (SVD) shows that the first component is sufficient to explain the data [Figs. 7 ▸(*a*)–7(*c*)]. The temporal evolution of the data (right singular vector) is relatively well described by the sum of one or two exponential functions. The time-constants were determined by minimizing the sum of squares of the residual between model and data and are found in Table 1 ▸. It should be noted that modest T-jumps somewhat shift the equilibrium between structural states, but structural transitions are still likely bidirectional. More thorough modelling should thus take into account that forward and reverse rates can be expected to be of similar magnitude.

The difference scattering signal (left singular vector, LSV) is virtually identical for all measurements [Fig. 7 ▸(*g*)]. These are clear indications that, regardless of whether the initial temperature is 20°C, 30°C or 40°C, lysozyme undergoes the same structural change, but at a varying rate. From Fig. 6 ▸ we can also tell that the magnitude of the difference scattering signal is smaller at 40°C, compared with the two lower temperatures. The simplest explanation for this is that the population of protein structures is different at this temperature, with a larger fraction of proteins already showing the ‘high temperature’ structure.

#### 3.4.1. IR absorption by the protein and radiation damage

**Figure 8:**
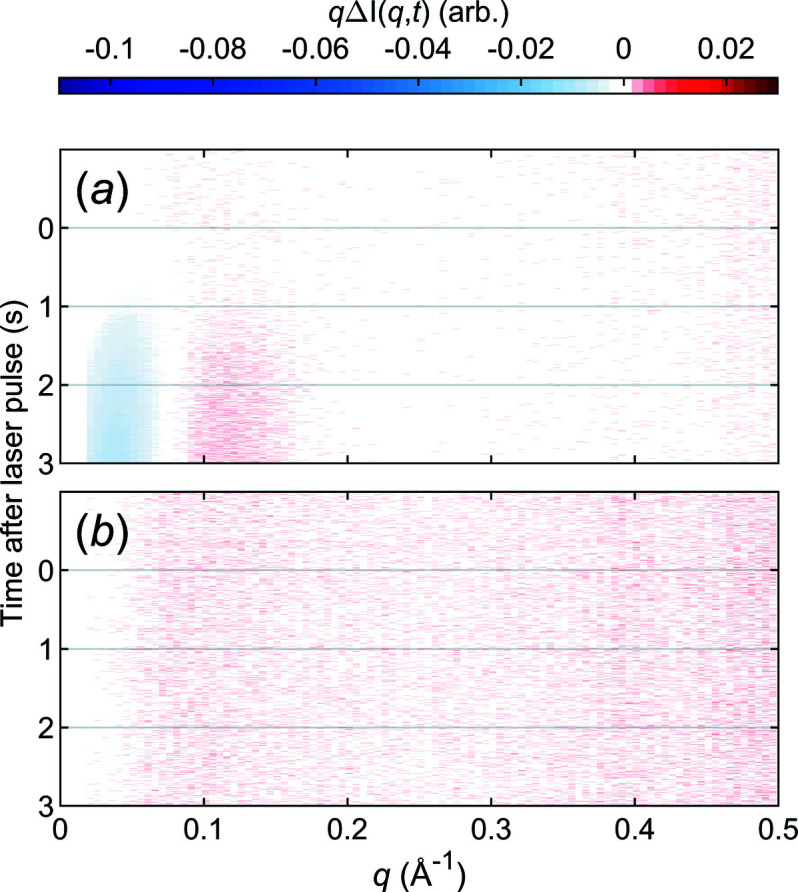
Data from a T-jump TR-XSS experiment on lysozyme. (*a*) Heat subtracted data in the SAXS range for a T-jump on lysozyme in deuterated buffer, starting at 20°C. (*b*) *Laser off*–*laser off* difference data for a T-jump starting at 20°C. Note that the colour scale is the same as in Fig. 6 ▸ in order to enable comparisons of the two.

Although the energy of 1470 nm light is primarily absorbed by water, proteins are not fully transparent to it. To assess whether IR radiation absorbed directly by the protein would have a significant impact on the experiment, we performed measurements of lysozyme in deuterated buffer. In Fig. 8 ▸(*a*) T-jump TR-XSS data on lysozyme in a deuterated buffer are shown. Compared with lysozyme in regular water the signal is essentially identical in shape but about an order of magnitude smaller. We attribute this signal primarily to partial exchange in the deuterated buffer as neither the protein nor the buffer components, apart from the water, was deuterated. We therefore conclude that IR radiation absorbed directly by the protein has little to no effect on the structure of the protein, as observed in SAXS.

During the experiment, the sample is held static in the X-ray beam for four seconds. This is bound to cause some radiation damage and this is also readily detected in the conventional SAXS data treatment. However, the use of the difference data protocol alleviates this. Fig. 8 ▸(*b*) shows difference data generated by subtracting *laser off* steps from each other. This shows that radiation damage affects all curves in the same way and this cancels out. Therefore it will also not matter if the protein in addition to the X-ray radiation was subjected to a temperature increase – the amount of radiation damage will still be the same and cancel out. This is based on the assumption that damaged proteins will not undergo a concerted structural change in response to a T-jump.

## 4. Summary and outlook

We have developed a setup at CoSAXS that can be used for TR-XSS measurements, with primary focus on millisecond T-jump experiments in aqueous environments. The setup makes use of the rapid frame rate and low background levels of the Eiger2 and Mythen2 detectors.

We have demonstrated that a fast, but relatively simple, detector like the Mythen2 1K can be used to monitor the changes in WAXS of water in response to temperature changes of ∼5–20°C. We have also demonstrated how a ∼15°C T-jump can be triggered by a 2 ms laser pulse and maintained for several seconds, by additional laser pulses. To our knowledge, maintaining a T-jump is a unique feature of the setup at CoSAXS. We have also characterized the spatial profile of the laser triggered T-jump in the capillary. This was done by simulations as well as by experiments, with very good correlation.

In addition, we have presented data that show that IR radiation absorbed directly by the protein does not have a pronounced effect compared with that caused by the temperature change of the solvent. Furthermore, the data presented also shows that, by calculating difference scattering, radiation damage cancels out. Assuming that only non-damaged proteins undergo a coordinated structural change, difference scattering [Δ*I*(*q*)] is less sensitive to radiation damage, compared with conventional SAXS [*I*(*q*)]. While it was not possible at the time of the experiments to properly test the underlying assumptions, we would like to propose a strategy by which it can be verified. By comparing the difference scattering signal at a defined time after the laser pulse whilst allowing the time from the onset of X-ray exposure to the primary laser pulse (Δ*t*_1_) to vary, it would be possible to assess what effect, if any, radiation damage has on the difference scattering data. We suggest that this should be verified for any system/sample under study.

At the time of writing, further developments of the CoSAXS instrument are already ongoing. During the fall of 2021 an L-shaped 2D Pilatus3 (Dectris) area detector was commissioned as the primary WAXS detector at CoSAXS. Apart from offering a continuous SAXS and WAXS range it will also make it possible to record data that require 2D detection, such as anisotropic samples.

Work is also in progress to make use of the gated mode of the detectors (Ejdrup *et al.*, 2009 ▸). In combination with the use of a nanosecond laser source, which is also being installed, this will make it possible to probe the structural response to T-jumps with a microsecond time-resolution.

Together, this would make CoSAXS a beamline with TR-XSS capabilities from the microsecond to second range, making it highly relevant for a wide range of biological samples.

## Supplementary Material

Supporting Figure S1. DOI: 10.1107/S1600577522000996/ay5592sup1.pdf

## Figures and Tables

**Table 1:** Derived parameters for the different lysozyme measurements in an H_2_O buffer solution (except where indicated)

*T*_init_	20°C	30°C	40°C	20°C (D_2_O)
T-jump (°C)	13.1 ± 1.1	15.8 ± 1.4	12.7 ± 1.3	0.5 ± 0.8
Time constant (s)	4.46 ± 0.53	5.26 ± 0.71	0.04 ± 0.01	–
			0.29 ± 0.023	
