# Supplementary material for: A setup for millisecond time-resolved X-ray solution scattering experiments at the CoSAXS beamline at the MAX IV Laboratory
Source: J Synchrotron Radiat. 2022 Feb 16;29(Pt 2):555–62. doi: 10.1107/S1600577522000996 (PMC8900842; doi:10.1107/S1600577522000996)
Supplement: Supplementary file 1 [file s-29-00555-sup1.pdf]

JOURNAL OF SYNCHROTRON RADIATION

Volume 29 (2022)

Supporting information for article:

A setup for millisecond time-resolved X-ray solution scattering experiments at the CoSAXS beamline at the MAX IV Laboratory

Oskar Berntsson, Ann E. Terry and Tomás S. Plivelic

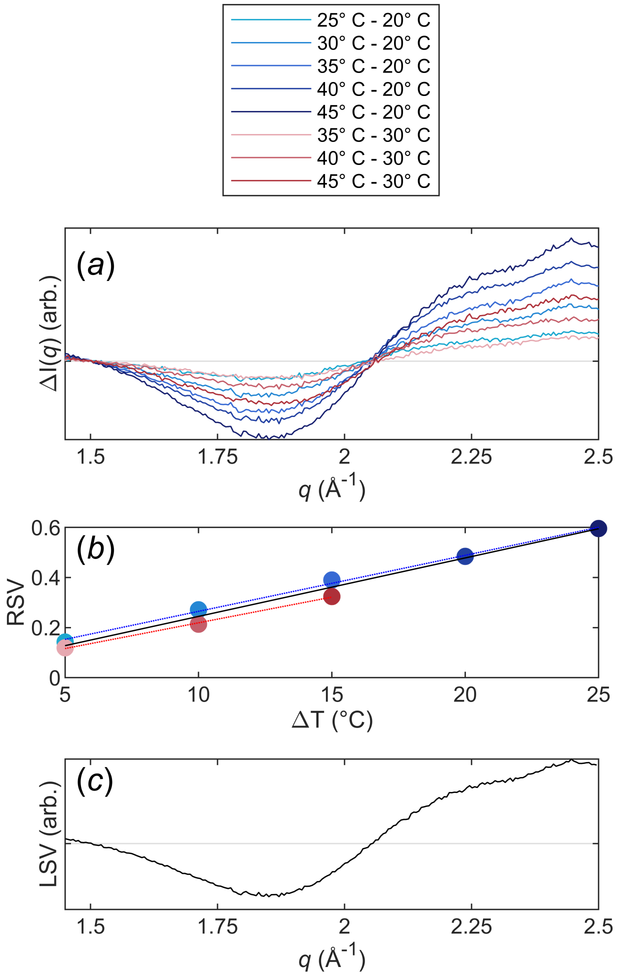

Fig. S1. WAXS measurements of water at different temperatures. (a) Difference scattering curves generated by subtracting data from static measurements at different temperatures. Blue curves use 20 °C as a reference temperature and red curves use 30 °C as a reference temperature. (b-c) The result of an SVD analysis where (b) shows the first RSV against temperature difference and (c) shows the first LSV.
